# STEAP4–Mediated ROS–TERT–TP53 Signaling Promotes Granulosa Cell Dysfunction in Experimental Models of Polycystic Ovary Syndrome

**DOI:** 10.3390/cells15131220

**Published:** 2026-07-04

**Authors:** Xinxin Quan, Xue Xue, Huilan Ma, Lei Yang, Chen Chen, Yu Liu, Kejie Yao, Hui Yang, Rongxiang Wang, Liya Shi, Lun Suo, Qiuju Chen, Lihua Sun

**Affiliations:** 1 Department of Cardiovascular Medicine, Shanghai East Hospital, Tongji University School of Medicine, Shanghai 200120, China; shsqxx@126.com (X.Q.); twinsnow@126.com (X.X.); mahuilan88@163.com (H.M.); 2205261@tongji.edu.cn (L.Y.); breeze-cici@163.com (C.C.); liuyu555555@126.com (Y.L.); ykj11382@163.com (K.Y.); hui_yang8836@163.com (H.Y.); wangrongxiang001@126.com (R.W.);; 2 Research Center for Translational Medicine, Shanghai East Hospital, Tongji University School of Medicine, Shanghai 200120, China; 3 Key Laboratory of Arrhythmias, Ministry of Education, Shanghai East Hospital, Tongji University School of Medicine, Shanghai 200120, China; 4 Department of Assisted Reproduction, Shanghai Ninth People’s Hospital, Shanghai Jiaotong University School of Medicine, Shanghai 200011, China

**Keywords:** PCOS, STEAP4, oxidative stress, TERT, P53 pathway

## Abstract

**Background:** Polycystic ovary syndrome (PCOS) is a frequently encountered endocrine disturbance with a still poorly defined etiology that arises in women during their reproductive years. Increased apoptosis of granulosa cells has been identified as one of the key factors contributing to abnormal follicular development. This study aimed to elucidate the role of six-transmembrane epithelial antigen of prostate 4 (STEAP4) in granulosa cell function using in vitro and in vivo models relevant to PCOS. **Methods:** We treated KGN cells (a human granulosa-like cell line) and C57BL/6 mice with dehydroepiandrosterone (DHEA) to establish experimental models mimicking PCOS features. STEAP4 expression was assessed by qRT–PCR, Western blot, and immunohistochemistry. Proliferative capacity and apoptotic rates were gauged with CCK-8 assays, EdU labeling, and flow cytometry. The regulatory mechanisms were investigated through immunofluorescence staining for nuclear factor erythroid–2–related factor 2 (Nrf2) nuclear translocation and immunoprecipitation assays for HIF-1α ubiquitination. **Results:** Exposure to androgen markedly raised both STEAP4 transcript and protein abundance in KGN cells as well as in PCOS model mice. STEAP4 knockdown resulted in increased proliferation and reduced apoptosis in DHEA–treated KGN cells. Mechanistically, STEAP4 enhanced reactive oxygen species levels, promoted Nrf2 nuclear translocation, and stabilized HIF–1α protein by reducing its ubiquitination, leading to increased TERT expression and subsequent TP53 pathway activation. In vivo, STEAP4 silencing significantly alleviated hormonal imbalances, estrous cycle disorders, and reduced oxidative stress levels in ovarian tissue of DHEA-induced PCOS–like mice. **Conclusions:** Taken together, evidence from these experimental models indicates that STEAP4 shapes oxidative stress and granulosa cell apoptosis by operating through the ROS–TERT–TP53 axis. The data point to a possible contribution of STEAP4 to PCOS pathogenesis and mark it as a candidate therapeutic target that merits additional clinical study.

## 1. Introduction

Polycystic ovary syndrome (PCOS) represents a common endocrine–gynecological condition that impacts 5–18% of women of childbearing age and constitutes a leading cause of female infertility globally [1,2]. Hyperandrogenism, ovulatory dysfunction, and multicystic ovaries constitute the core diagnostic features of PCOS [3]. In addition, patients with PCOS often present with metabolic disorders, such as insulin resistance, obesity, and abnormal lipid metabolism, which lead to an increased risk of type 2 diabetes, cardiovascular disease, and endometrial cancer [3,4]. The clinical management of PCOS presently centers on controlling clinical manifestations and reducing future health risks, and include lifestyle changes such as diet, sleep, exercise and stress reduction, as well as pharmacotherapeutic interventions such as anti-androgens or metformin, which manage symptoms associated with irregular periods, acne and hair growth [5,6,7]. Nevertheless, because the underlying cause of PCOS has yet to be defined, designing more effective treatments for affected women will depend on a fuller grasp of how the disease develops.

A complex etiology underlies PCOS, with genetic, epigenetic, environmental and lifestyle contributors all implicated [8]. More recently, granulosa cells, which alongside theca cells constitute the principal cellular elements of ovarian follicles, have emerged as important participants in the onset and progression of PCOS [9]. Granulosa cells provide essential nutrients, hormones, growth factors and structural support that are critical for oocyte maturation [10]. However, diminished proliferation together with heightened apoptosis of granulosa cells has been linked to retarded follicular growth and the resulting build–up of immature follicles within the ovaries of women with PCOS [11]. Clarifying how granulosa cell proliferation and apoptosis operate in PCOS may therefore inform the design of new treatment approaches.

The metalloreductase six–transmembrane epithelial antigen of prostate 4 (STEAP4) serves as a key regulator of both iron and copper homeostasis [12], as well as cell cycle regulation, proliferation, differentiation, and apoptosis [13,14]. First described in 2005, the six-transmembrane protein STEAP4 (originally termed STAMP2) is regulated by androgens and was found to be upregulated in prostate cancer biospecimens [15]. Subsequent work has connected STEAP4 to a range of conditions, among them several cancers, insulin resistance, non-alcoholic fatty liver disease, and benign prostatic hyperplasia [16]. Given that PCOS features elevated androgens and that STEAP4 is induced upon androgen receptor activation [17], we reasoned that STEAP4 might participate in the pathogenesis of PCOS.

In this work, we set out to define the contribution of STEAP4 to PCOS–associated granulosa cell dysfunction with the use of experimental models. We showed that STEAP4 was upregulated in ovarian tissue samples obtained from PCOS patients as well as in DHEA–treated KGN cells and mouse ovarian tissues. We further showed that STEAP4 knockdown enhances proliferation while lowering apoptosis in DHEA–stimulated granulosa cells. In addition, we found that STEAP4 increases oxidative stress, which facilitates nuclear factor erythroid–2–related factor 2 (Nrf2) nuclear translocation and stabilizes hypoxia-inducible factor-1alpha (HIF–1α) protein, thereby regulating telomerase reverse transcriptase (TERT). STEAP4–induced increases in TERT expression levels lead to P53 pathway activation in our experimental models. Overall, the present work links STEAP4 to defective granulosa cell function in PCOS and raises the possibility of targeting this molecule therapeutically, pending verification in clinical settings.

## 2. Materials and Methods

### 2.1. Patient Cohort and Bioinformatic Analysis

This investigation enrolled 30 PCOS cases fulfilling the Rotterdam consensus criteria, alongside 30 unaffected controls who reported no previous reproductive morbidity, who were admitted to the Shanghai East Hospital, Tongji University School of Medicine between March 2024 and October 2024. Transvaginal ultrasounds were performed on all patients by the same gynecologist using a 5–9 MHz transvaginal transducer (Voluson E8, GE Healthcare, Chicago, IL, USA). Polycystic ovarian morphology was defined as ovary volume > 10 cm^3^ and/or the presence of ≥12 follicles in the ovary. This study was conducted in accordance with the principles of the Declaration of Helsinki and was approved by the Ethics Committee of Shanghai East Hospital, Tongji University School of Medicine (license number 2022–015). Written informed consent was obtained from all patients prior to inclusion in this study. Bioinformatics analysis was carried out using the GEO datasets GSE226146 and GSE54250 to assess STEAP4 expression levels in patients with and without PCOS.

### 2.2. Cell Lines and Reagents

KGN, a steroidogenic human granulosa cell-like tumor line, was obtained from iCell Bioscience Inc. (Shanghai, China). Cells were maintained in Dulbecco’s modified Eagle medium F–12 supplemented with 10% fetal bovine serum and penicillin/streptomycin (100 units/mL) at 37 °C and an atmosphere of 5% CO_2_. A cellular model of PCOS was established by treating KGN cells with 20 μM dehydroepiandrosterone (DHEA; Macklin, Shanghai, China) for 48 h [18].

### 2.3. Cell Transfection

shRNAs specific to STEAP4 and TERT were designed using the Sigma-Aldrich online tool (https://www.sigmaaldrich.cn/) and synthesized by Genechem (Shanghai, China). The target sequences of the shRNAs used in this study were as follows: human sh–STEAP4#1 (5′-CCAAGAAGTCTGACATCATAA-3′), human sh–STEAP4#2 (5′-GCAGGTGTTTGTGTGTGGAAA-3′), human sh–TERT#1 (5′-GAAGAGTGTCTGGAGCAAGTT-3′), human sh–TERT#2 (5′-GCATTGGAATCAGACAGCACT-3′), mouse sh–STEAP4 (5′-GTTCAGTCCAAACTGGGTTAT-3′), and scrambled control sh–NC (5′-TTCTCCGAACGTGTCACGT-3′).

KGN cells were seeded in 6-well plates at 70% confluence and transfected with shRNAs (20 μM) or scrambled control (sh–NC) using Lipofectamine 2000 (Invitrogen, Carlsbad, CA, USA) according to the manufacturer’s protocol. Briefly, shRNA and Lipofectamine 2000 were separately diluted in Opti-MEM medium, combined, and incubated for 20 min at room temperature before adding to cells. Following a 6-h exposure to the transfection mixture, the culture medium was discarded and replenished with normal growth medium. At 48 h after transfection, the cells were collected for downstream assays.

TERT overexpression plasmids were constructed by cloning human TERT cDNA into pCMV6-Entry vector (RC217436, Origene, Rockville, MD, USA) for human cell experiments, and mouse TERT cDNA into pCMV6-Entry vector (MR226892, Origene) for mouse experiments. Overexpression plasmids were transfected using the same Lipofectamine 2000 protocol described above.

For in vivo experiments, shRNAs were cloned into lentiviral vectors by Genechem (Shanghai, China) to achieve stable knockdown. Lentiviral particles were produced with a titer of 1 × 10^8^ TU/mL and used for ovarian injection.

### 2.4. Dual-Luciferase Reporter Gene Assay

293T cells were plated and transfected in triplicate using Lipofectamine 2000 (Invitrogen, USA), following the recommended protocol, with the luciferase vector plus the specified transcription factors. The dual–luciferase reporter assay (Promega, Madison, WI, USA) was employed to evaluate luciferase activity at 48 h after transfection, and normalization was performed against Renilla luciferase activity.

### 2.5. Cell Counting Kit-8 (CCK-8) Assay

The CCK8 assay (Beyotime, Shanghai, China) was used to determine cell viability following the supplier’s protocol. Briefly, KGN cells were plated into 96-well plates and allowed to adhere for 24 h. Following DHEA and shRNA treatment, the cells received CCK-8 solution for a 2 h incubation at 37 °C. Optical density was read at 450 nm on a microplate reader (Thermo Fisher Scientific, Waltham, MA, USA). Each experiment was carried out in triplicate and repeated on three separate occasions.

### 2.6. Flow Cytometry Analysis

Levels of apoptosis were determined by flow cytometry. Briefly, cells cultured in 6-well plates received different experimental interventions. Cells were harvested using EDTA-free trypsin, washed twice in PBS, then centrifuged at 1000× *g* for 5 min. The cell pellet was resuspended in Annexin V Binding Buffer (500 μL; Thermo Fisher Scientific, Waltham, MA, USA). Annexin V–FITC (5 μL; Thermo Fisher Scientific) and propidium iodide (PI; 5 μL; Thermo Fisher Scientific) were added and samples were incubated for 10 min at room temperature in the dark. Apoptotic cells were detected using a flow cytometer (BD Pharmingen, Franklin Lakes, NJ, USA).

### 2.7. EdU Staining

Levels of cell proliferation were determined using an EdU Kit (#C0071S, Beyotime). Briefly, cells were seeded into 24-well plates and incubated for 24 h. Following treatment with DHEA and shRNAs, cells were incubated with EdU solution for 4 h at 37 °C and fixed in paraformaldehyde for 15 min. Cells were then stained with the Apollo dye reagent for 30 min and nuclei were stained with 4′,6–diamidino–2–phenylindole (DAPI) for 5 min. Samples were visualized by fluorescence microscopy (Olympus, Tokyo, Japan). Experiments were repeated three times in triplicate.

### 2.8. Measurement of Intracellular and Mitochondrial ROS Levels

Intracellular and mitochondrial ROS levels were determined using 2′,7′-dichlorodihydrofluorescein diacetate (DCFH–DA, #S0033S, Beyotime) and MitoSOX^TM^ Red (M36008, Thermo Fisher Scientific) fluorescence probes, respectively. KGN cells were harvested, washed three times in PBS, then incubated with 5 μM DCFH–DA–A or MitoSOXTM Red for 45 min at 37 °C in the dark. Relative levels of fluorescence were quantified using a flow cytometer (CytoFLEX, Beckman Coulter, Brea, CA, USA). Data were analyzed using FlowJo V10 software.

### 2.9. Oxidative Stress Detection

KGN cells and mouse ovarian tissue samples were lysed and homogenized, respectively. Samples were centrifuged at 10,000× *g* for 10 min, and the supernatants were collected. Superoxide dismutase (SOD), malondialdehyde (MDA), and glutathione (GSH) levels were measured using the Total Superoxide Dismutase Assay Kit with NBT, Lipid Peroxidation MDA Assay Kit, and Glutathione Assay Kit (Solarbio, Beijing, China), respectively. Experiments were repeated three times in triplicate.

### 2.10. Lipid Peroxidation Assay

KGN cells were seeded into 12–well plates and subjected to various treatments. Intracellular lipid peroxidation levels were assessed by incubating treated cells with serum-free medium containing 2 μM BODIPY 581/591C11 dye (HY–D1301, MedChemExpress, Shanghai, China) for 30 min at 37 °C in the dark. Cells were then harvested by trypsinization, washed three times with PBS and resuspended in PBS. Lipid peroxidation signals were detected by flow cytometry and analyzed using FlowJo V10 software.

### 2.11. Immunofluorescence Staining

Control or DHEA-treated KGN cells were fixed in 100% methanol for 5 min, then lysed by incubating with 0.5% Triton X–100 for 30 min. Samples were blocked with 10% normal goat serum for 30 min, then incubated with a primary antibody against Nrf2 (#12721, 1:500, Cell signaling Technology, Danvers, MA, USA) overnight at 4 °C. After washing with PBS, cells were incubated with the corresponding fluorescence–labeled secondary antibody for 30 min at room temperature in the dark. Nuclear DNA was stained with DAPI. Samples were sealed with a sealing solution containing anti–fluorescence quenching agent, and Nrf2 nuclear translocation was observed under a fluorescence microscope (Nikon Eclipse 400, Nikon Corporation, Kanagawa, Japan).

### 2.12. Establishment of a Mouse Model of PCOS

All animal procedures were approved by the Ethics Committee of Shanghai East Hospital, Tongji University School of Medicine and conducted in accordance with the Guide for the Care and Use of Laboratory Animals. Three–week–old female C57BL/6J mice (9–12 g) were purchased from Beijing Vital River Laboratory Animal Technology Co., Ltd. (Beijing, China). Mice were maintained in a pathogen-free environment with controlled temperature (25 °C) and light cycle (12 h light/12 h dark) with food and water ad libitum.

After one week of acclimatization, mice were randomized into five study arms (*n* = 6 mice per group): (1) vehicle control, (2) DHEA + sh–NC, (3) DHEA + sh–STEAP4, (4) DHEA + sh–STEAP4 + Vector, and (5) DHEA + sh–STEAP4 + TERT–OE. For lentiviral transduction, mouse–specific shRNAs targeting STEAP4 or negative control sequences, as well as mouse TERT overexpression constructs, were cloned into lentiviral vectors. Under isoflurane anesthesia, a small midline incision was made in the lower abdomen to expose the ovaries. Lentiviruses (10 μL, 1 × 10^8^ TU/mL) were carefully injected into the ovarian bursa bilaterally using a 30–gauge microsyringe (Hamilton, Reno, NV, USA). The specific treatments for each group were:

Vehicle group: sham surgery without viral injection;

DHEA + sh–NC group: lentivirus carrying scrambled shRNA control;

DHEA + sh–STEAP4 group: lentivirus carrying mouse STEAP4–specific shRNA;

DHEA + sh–STEAP4 + Vector group: co-injection of STEAP4 shRNA lentivirus and empty vector control;

DHEA + sh–STEAP4 + TERT–OE group: co-injection of STEAP4 shRNA lentivirus and mouse TERT overexpression lentivirus.

The microsyringe was held in place for 5–7 min after injection to prevent viral backflow. The peritoneum and skin were sutured in layers, and mice received post-operative analgesia. Animals were allowed to recover for 5 days before PCOS induction.

Following recovery, PCOS was induced by daily subcutaneous injection of DHEA (60 mg/kg/day, dissolved in 0.1 mL sesame oil) for 21 consecutive days. PCOS groups were simultaneously switched to a high-fat diet (60% fat, 14.1% protein, 25.9% carbohydrate, energy density: 5 kcal/g; TROPHIC Animal Feed High-Tech Co., Ltd., Shanghai, China, TP23400) throughout the treatment period. The vehicle control group received daily subcutaneous injections of 0.1 mL sesame oil and was maintained on standard chow diet (10.0% water, 18.0% crude protein, 4.0% crude fat, 5.0% crude fiber, 8.0% crude ash; Beijing Hfk Bioscience Co., Ltd., Beijing, China).

Body weight was recorded daily at 9:00 AM to calculate accurate DHEA dosage. Vaginal smears were collected every morning at 9:00 AM throughout the 21–day treatment period to assess estrous cycle stages. Smears were obtained by gently flushing the vaginal cavity with 20 μL of 0.9% saline, spread on glass slides, air-dried, and stained with Giemsa solution. The estrous cycle stage was determined by microscopic examination of the predominant cell types: nucleated epithelial cells (proestrus), cornified epithelial cells (estrus), cornified epithelial cells with leukocytes (metestrus), and predominantly leukocytes (diestrus).

After 21 days of treatment, mice were fasted overnight and humanely euthanized by cervical dislocation under deep isoflurane anesthesia. Blood samples were immediately collected from the abdominal aorta using heparinized syringes for serum hormone analysis. Both ovaries were rapidly dissected and weighed. The right ovary was fixed in 4% paraformaldehyde for 24 h for histological and immunohistochemical analyses, while the left ovary was either snap–frozen in liquid nitrogen for molecular studies or used immediately for granulosa cell isolation.

### 2.13. Estrous Cycle Assessment

The estrous cycle comprises four phases, namely, proestrus, estrus, metestrus, and diestrus [19]. Different phases of the estrous cycle were assessed by collecting samples from the vaginas of mice. Slides were prepared, stained with Giemsa stain, and vaginal cytology was evaluated using light microscopy (Leitz Orthoplan, Leitz, Wetzlar, Germany).

### 2.14. Hormone Analysis

Blood samples were collected from the abdominal aorta of mice in each treatment group and centrifuged at 14,000× *g* for 10 min at 4 °C to obtain serum. Serum samples were aliquoted and stored at −80 °C until analysis. Serum hormone levels were measured using commercially available ELISA kits from SANGON BIOTECH (Shanghai, China) according to the manufacturer’s instructions. Serum testosterone levels were determined using a mouse testosterone ELISA kit (D721374). Follicle-stimulating hormone (FSH) concentrations were measured using a mouse FSH ELISA kit (D721074). Luteinizing hormone (LH) levels were quantified using a mouse LH ELISA kit (D721182). Anti-Müllerian hormone (AMH) concentrations were detected using a mouse AMH ELISA kit (D721203). Progesterone levels were measured using a mouse progesterone ELISA kit (D721377). All samples were analyzed in duplicate, and hormone concentrations were calculated based on standard curves generated for each assay. The intra- and inter-assay coefficients of variation were less than 10% for all assays.

### 2.15. Measurement of Fasting Glucose and Insulin Levels

Mice were fasted overnight, then blood glucose and plasma insulin levels were measured using an Accu-Chek^®^ glucometer (Roche Diagnostics, Indianapolis, IN, USA) and the Ultra-Sensitive Mouse Insulin ELISA Kit (Crystal Chem, Downers Grove, IL, USA), respectively. The homeostatic model assessment of insulin resistance (HOMA-IR) index was calculated using the formula: [fasting glucose levels (mmol/L)] × [fasting serum insulin (mulU/mL)]/22.5.

### 2.16. Hematoxylin and Eosin (H&E) Staining and Immunohistochemistry (IHC)

Ovarian tissue samples were fixed in 4% buffered formalin overnight, then embedded in paraffin. The paraffin blocks were then sectioned and stained with H&E using the Hematoxylin-Eosin Staining Kit (MCE, HY-K0315, Shanghai, China).

IHC staining was performed on paraffin-embedded ovarian tissue sections (4 μm thick). After deparaffinization and rehydration, antigen retrieval was carried out in citrate buffer (10 mM, pH 6.0) using microwave heating. Endogenous peroxidase activity was blocked with 3% H_2_O_2_ for 10 min, followed by blocking with 5% normal goat serum for 1 h at room temperature. Sections were then incubated with primary antibodies against STEAP4 (1:500, Proteintech, 11944–1–AP, Rosemont, IL, USA) and Ki-67 (1:500, Abcam, ab15580, Cambridge, UK) overnight at 4 °C. After washing, sections were incubated with HRP-conjugated secondary antibody (1:2000, Beyotime, Shanghai, China) for 1 h at room temperature. Immunoreactivity was visualized using a DAB Staining Kit (Beyotime), followed by hematoxylin counterstaining. For each sample, 3–5 random fields at 40× magnification was captured using a light microscope (Olympus, Tokyo, Japan). Quantitative analysis was performed using Image Pro-Plus 6.0 software (Media Cybernetics, Rockville, MD, USA).

### 2.17. TUNEL Assay

Apoptosis was assessed in mouse ovarian tissue using the One Step TUNEL Apoptosis Detection Kit (FITC) (Beyotime Biotechnology, #C1088). Briefly, tissue slices were de-paraffinized, rehydrated through graded alcohols (100%, 95%, 85%, 70%), then incubated with proteinase K (20 μg/mL) for 20 min at room temperature. After washing with PBS three times, samples were incubated with 3% H_2_O_2_ solution for 10 min to inactivate endogenous peroxidase, then washed in PBS again. Tissues were then permeabilized with 0.1% Triton X–100 in 0.1% sodium citrate for 8 min on ice. After washing, sections were incubated with TUNEL reaction mixture (TdT enzyme and fluorescein-labeled dUTP) for 60 min at 37 °C in a humidified chamber in the dark. Following three washes with PBS, nuclei were counterstained with DAPI (1 μg/mL) for 5 min. Slides were mounted with anti-fade mounting medium and visualized using fluorescence microscopy (Olympus, Tokyo, Japan). TUNEL–positive cells were counted in five random fields per section and expressed as a percentage of total cells.

### 2.18. Quantitative Real-Time Polymerase Chain Reaction (qRT-PCR)

Total RNA was extracted from cell and tissue samples using TRIzol reagent (Invitrogen, Carlsbad, CA, USA) according to the manufacturer’s protocol. RNA concentration and purity were measured using a spectrophotometer (NanoDrop 2000, Thermo Fisher Scientific, Waltham, MA, USA). Reverse transcription was performed using a PrimeScript RT reagent kit (TaKaRa Bio, Otsu, Japan) to synthesize cDNA from total RNA. Quantitative real-time PCR (qRT–PCR) was then carried out using SYBR Premix Ex Taq (TaKaRa Bio, Otsu, Japan) on a real-time PCR system. The thermal cycling conditions were as follows: initial denaturation at 95 °C for 30 s, followed by 40 cycles of 95 °C for 5 s and 60 °C for 34 s. Glyceraldehyde phosphate dehydrogenase (GAPDH) was used as an internal control. Relative mRNA expression levels were calculated using the 2^−ΔΔCt^ method. All reactions were performed in triplicate. The primer sequences for all tested genes are listed in Table 1.

### 2.19. Western Blotting

Cells were collected, washed with ice-cold PBS, and total protein was extracted using RIPA lysis buffer (Beyotime, Shanghai, China) supplemented with protease inhibitors. Protein concentrations were determined using a BCA assay kit (Beyotime, Shanghai, China). Equal amounts of protein were separated by SDS-PAGE and transferred onto PVDF membranes (Millipore, Billerica, MA, USA). Membranes were blocked with 5% non-fat milk for 1 h at room temperature, then incubated overnight at 4 °C with primary antibodies (listed in Table 2). After washing, membranes were incubated with HRP-conjugated secondary antibody for 1 h at room temperature. Protein bands were visualized using an ECL kit (Thermo Fisher Scientific, #32209, Waltham, MA, USA) and quantified using ImageJ software (version 1.8.0). GAPDH was used as the loading control.

### 2.20. Statistical Analysis

Data analysis was carried out using GraphPad Prism 9 (GraphPad Software, San Diego, CA, USA). Normal distribution was validated using the Shapiro–Wilk test. The correlation between STEAP4 and TERT expression levels was determined using the Pearson correlation analysis. Data are presented as mean ± standard deviation (SD). Data between two groups were compared using the independent *t* test, while differences between multiple groups were analyzed using one-way analysis of variance followed by Tukey’s test. *p* values were obtained by two-sided tests, and *p* < 0.05 was considered statistically significant.

## 3. Results

### 3.1. STEAP4 Is Upregulated in Granulosa Cells of PCOS Patients and DHEA–Induced PCOS-like Mice

**Figure 1:**
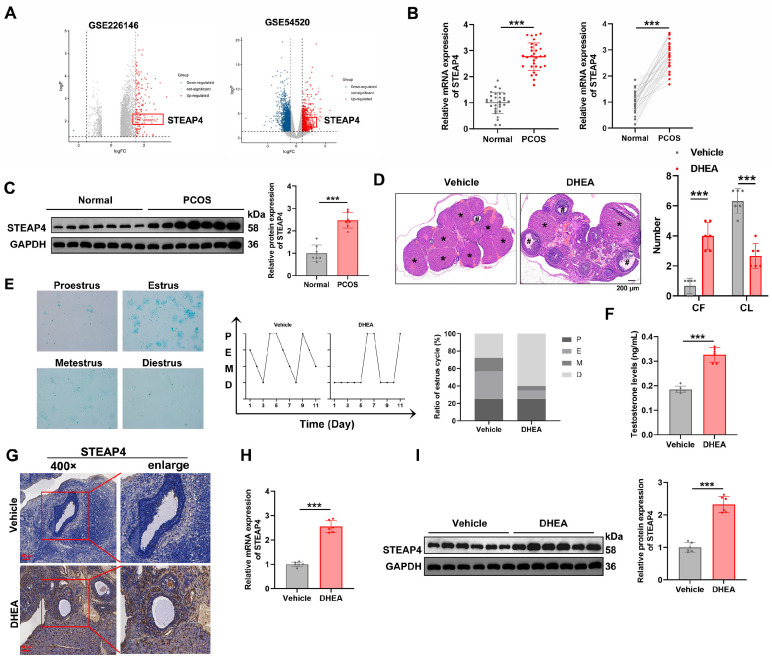
Elevated STEAP4 expression in granulosa cells of PCOS patients and DHEA–induced PCOS–like mice. (**A**) Analysis of STEAP4 expression in granulosa cells from public GEO datasets (GSE226146 and GSE54250) showing increased STEAP4 levels in PCOS patients compared to controls. (**B**) qRT–PCR validation of STEAP4 mRNA expression in granulosa cells isolated from PCOS patients (*n* = 30) and control subjects (*n* = 30). GAPDH was used as internal control. Data passed normality test (Shapiro–Wilk test) and statistical analysis was performed using unpaired *t*-test. (**C**) Western blot analysis of STEAP4 protein levels in granulosa cells from PCOS patients (*n* = 7) and control subjects (*n* = 7). GAPDH served as loading control. Representative blots (left) and quantification (right) are shown. Statistical analysis was performed using unpaired *t*-test. (**D**) H&E staining morphology of ovarian tissues from DHEA–induced mice (*n* = 6) and their control (*n* = 6). #: cystic follicles (CF). *: corpora lutea (CL). Scale bar = 200 μm. (**E**) Vaginal cytology assessment of estrous cycles for 11 consecutive days. P, proestrus; E, estrus; M, metestrus; D, diestrus. (**F**) ELISA detection of serum testosterone (T) of DHEA-induced mice and control, *n* = 6. (**G**) Immunohistochemical staining of STEAP4 in ovarian sections from DHEA–induced PCOS mice and control mice. Scale bar = 50 μm. Quantification of STEAP4 staining intensity (right) was performed using ImageJ software, *n* = 6. (**H**) qRT–PCR validation of STEAP4 mRNA expression in granulosa cells isolated from DHEA–induced mice and control subjects, *n* = 6. GAPDH was used as internal control. (**I**) Western blot analysis of STEAP4 protein expression in granulosa cells isolated from DHEA–induced mice and control subjects, *n* = 6. GAPDH was used as internal control. Data are presented as mean ± SD. Statistical analysis was performed using one–way ANOVA followed by Tukey’s post hoc test. *** *p* < 0.001.

As a first step toward defining how STEAP4 contributes to PCOS, we surveyed its expression in granulosa cells. Two independent GEO datasets (GSE226146 and GSE54250) showed significantly higher STEAP4 in PCOS granulosa cells than in controls (Figure 1A), and in a validation cohort of 30 PCOS patients and 30 age–matched controls this was confirmed at both the mRNA (Figure 1B) and protein (Figure 1C) levels. We next examined DHEA–induced PCOS–like mice, which displayed an increased number of cystic follicles (Figure 1D) together with abnormal estrous cycles and elevated serum T (Figure 1E,F), confirming successful model establishment. IHC showed STEAP4 to be localized predominantly in follicular granulosa cells, with markedly stronger staining in DHEA–induced PCOS ovaries than in controls (Figure 1G), while qRT–PCR and Western blotting revealed 2.4–fold and 2.3–fold increases in STEAP4 mRNA and protein, respectively, in the PCOS group (Figure 1H,I). Together, these data demonstrate that STEAP4 is consistently upregulated, at both mRNA and protein levels, in granulosa cells under hyperandrogenic conditions.

### 3.2. STEAP4 Knockdown Rescues DHEA-Induced Inhibition of Proliferation and Promotion of Apoptosis in KGN Cells

**Figure 2:**
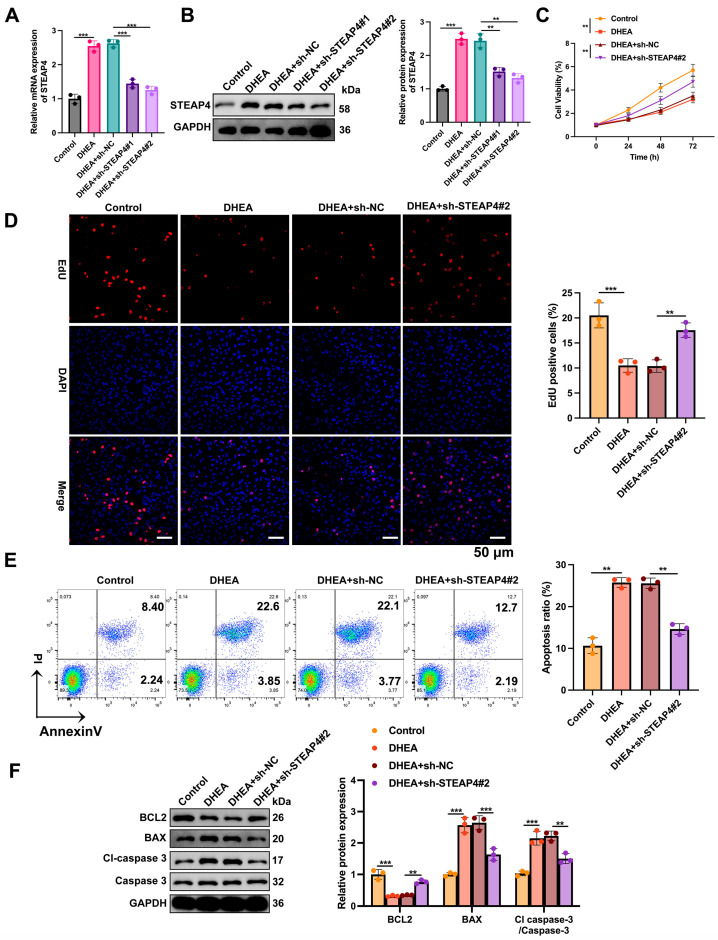
Effects of STEAP4 knockdown on DHEA-induced proliferation inhibition and apoptosis promotion in KGN cells. (**A**) qRT–PCR analysis of STEAP4 mRNA expression in KGN cells. Five groups were analyzed: Control, DHEA (20 μM, 48 h), DHEA + sh–NC, DHEA + sh–STEAP4#1, and DHEA + sh–STEAP4#2. GAPDH served as internal control. (**B**) Western blot analysis of STEAP4 protein expression in KGN cells with the same five treatment groups as in (**A**). GAPDH served as loading control. Representative blots (right) and quantification (left) are shown. (**C**) Cell viability assessed by CCK–8 assay at indicated time points. Cells were treated with DHEA and transfected with sh–STEAP4#2 or sh–NC as indicated. (**D**) Cell proliferation evaluated by EdU incorporation assay. Representative images (left) and quantification of EdU–positive cells (right) are shown. Scale bar = 50 μm. (**E**) Flow cytometry analysis of cell apoptosis using Annexin V–FITC/PI double staining. Representative plots (left) and quantification of apoptotic cells (right) are shown. (**F**) Western blot analysis of apoptosis-related proteins including BCL–2, BAX, caspase–3 and cleaved caspase–3. GAPDH served as loading control. Representative blots (left) and quantification (right) are shown. Data are presented as mean ± SD from three independent experiments. Statistical analysis was performed using one-way ANOVA followed by Tukey’s post hoc test. ** *p* < 0.01, *** *p* < 0.001.

For the in vitro work, we used KGN cells, a human granulosa cell line that, despite its granulosa cell tumor origin, retains granulosa cell–specific markers (FSHR, CYP19A1, AMH) and is a widely used cellular model for PCOS research [20,21]; their responses to hyperandrogenic conditions are concordant with those of primary human granulosa cells [22]. To mimic the hyperandrogenic environment of PCOS, KGN cells were treated with DHEA (20 μM) for 48 h, which raised STEAP4 at both the mRNA and protein levels relative to controls (Figure 2A,B). We then knocked down STEAP4 in DHEA–treated cells with two shRNAs (sh–STEAP4#1 and sh–STEAP4#2) and confirmed the efficiency by qRT-PCR and Western blotting. As sh–STEAP4#2 gave a stronger knockdown than sh–STEAP4#1 (Figure 2A,B), it was used for the subsequent functional assays. DHEA suppressed KGN cell proliferation, reflected by lower CCK–8 viability and fewer EdU–positive cells (Figure 2C,D), and induced apoptosis, with more apoptotic cells on flow cytometry (Figure 2E), reduced BCL–2, and elevated BAX and cleaved Caspase–3 (Figure 2F). STEAP4 knockdown reversed these effects: it restored viability and EdU incorporation toward control levels (Figure 2C,D), lowered the apoptotic fraction (Figure 2E), and normalized the apoptosis–related proteins (higher BCL–2, lower BAX and cleaved Caspase–3) versus the DHEA + sh–NC group (Figure 2F). STEAP4 is therefore a key mediator of DHEA–induced granulosa cell dysfunction, and its inhibition rescues both the proliferation defect and apoptosis.

### 3.3. Effects of STEAP4 Knockdown on Cellular Oxidative Stress and Related Phenotypes

**Figure 3:**
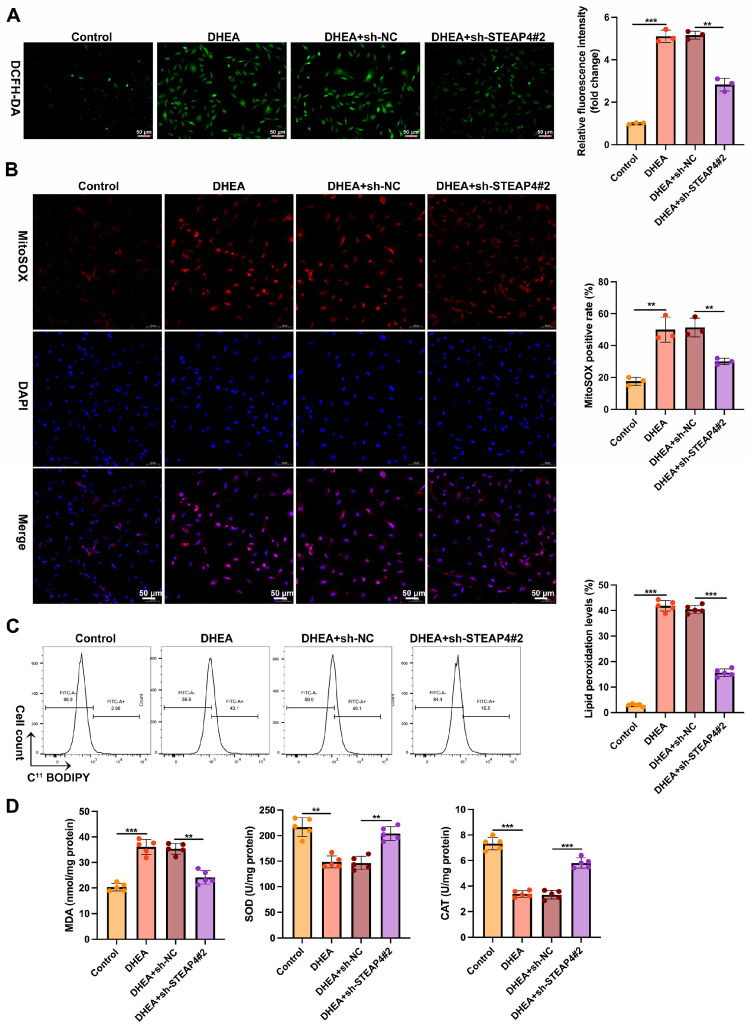
Effects of STEAP4 knockdown on oxidative stress parameters in DHEA–treated KGN cells. (**A**) Intracellular ROS levels measured by DCFH–DA staining in KGN cells. Four groups were analyzed: Control, DHEA (20 μM, 48 h), DHEA + sh–NC, and DHEA + sh–STEAP4#2. Representative fluorescence images (left) and quantification of fluorescence intensity (right) are shown. Scale bar = 50 μm, *n* = 3. (**B**) Mitochondrial ROS production detected using MitoSOX Red staining in the same four treatment groups. Representative images (left) and quantification (right) are shown. Scale bar = 50 μm, *n* = 3. (**C**) Lipid peroxidation levels detected using C11–BODIPY 581/591 probe by flow cytometry. Representative flow cytometry histograms (left) and quantification of lipid peroxidation levels (%) (right) are shown, *n* = 5. (**D**) Oxidative stress markers measured in KGN cells: MDA levels (left), SOD activity (middle), and CAT activity (right) in all four treatment groups, *n* = 5. Data are presented as mean ± SD. Statistical analysis was performed using one-way ANOVA followed by Tukey’s post hoc test. ** *p* < 0.01, *** *p* < 0.001.

Oxidative stress plays an important role in the development of PCOS [23,24,25]. Oxidative damage reportedly induces apoptosis in granulosa cells, ultimately affecting follicle quality [26]. Interestingly, STEAP4 has been shown to regulate cellular ROS levels in various cell types [27]. To assess whether STEAP4 regulates oxidative stress in granulosa cells, we first examined the effect of DHEA on cellular redox status. DHEA raised intracellular ROS (DCFH-DA, Figure 3A) and mitochondrial ROS (MitoSOX Red, Figure 3B), increased lipid peroxidation (C11-BODIPY 581/591, Figure 3C), and elevated MDA while lowering SOD and CAT activities relative to untreated controls (Figure 3D). STEAP4 knockdown with sh–STEAP4#2 reversed these changes: intracellular and mitochondrial ROS returned to near-control levels (Figure 3A,B), lipid peroxidation decreased (Figure 3C), and MDA fell while SOD and CAT activities recovered compared with the DHEA + sh–NC group (Figure 3D). STEAP4 is thus a key mediator of DHEA–induced oxidative stress in granulosa cells, and its inhibition restores redox homeostasis.

### 3.4. STEAP4 Regulates TERT Expression Through the ROS–Nrf2 Signaling Pathway

**Figure 4:**
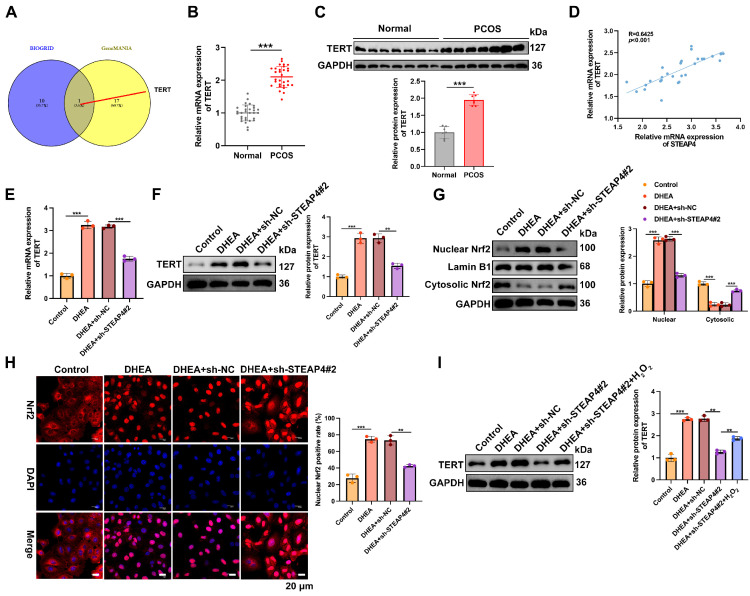
The ROS-Nrf2 axis mediates STEAP4–dependent TERT regulation in KGN cells. (**A**) Venn diagrams displaying the overlap of downstream targets of STEAP4 from BIOGRID and GeneMANIA databases. (**B**) qRT–PCR analysis of TERT mRNA expression levels in granulosa cells from PCOS patients (*n* = 30) and control subjects (*n* = 30). GAPDH served as the internal control. (**C**) Western blot analysis of TERT protein expression levels in granulosa cells from PCOS patients (*n* = 7) and control subjects (*n* = 7). GAPDH served as the loading control. (**D**) Correlation analysis between STEAP4 and TERT mRNA expression levels in granulosa cells from PCOS patients (*n* = 30) and controls (*n* = 30). qRT–PCR (**E**) and Western blot (**F**) analyses of TERT expression levels in DHEA–treated KGN cells transfected with sh-STEAP4#2 or sh–NC. (**G**) Western blot analysis of the nuclear and cytoplasmic expression levels of Nrf2 in DHEA-treated KGN cells after STEAP4 knockdown. Lamin B1 and GAPDH served as the nuclear and cytoplasmic loading controls, respectively. (**H**) Immunofluorescence staining showing Nrf2 nuclear translocation in DHEA–treated KGN cells transfected with sh–STEAP4#2 or sh–NC. Scale bar = 20 μm. (**I**) Western blot analysis of TERT expression levels in STEAP4–knockdown DHEA–treated KGN cells treated with or without H_2_O_2_ (150 μM) for 24 h. Data are presented as mean ± SD. ** *p* < 0.01, *** *p* < 0.001.

To identify potential downstream targets of STEAP4, we performed bioinformatics analysis using BIOGRID and GeneMANIA databases, which revealed TERT as a potential downstream effector of STEAP4 (Figure 4A). Earlier work has demonstrated that Nrf2 can promote TERT expression and govern the pentose phosphate pathway in glioblastoma [28], as well as attenuate ferroptosis in acute lung injury by modulating TERT and SLC7A11 expression [29]. It is well established that oxidative stress promotes Nrf2 nuclear translocation by disrupting the KEAP1–Nrf2 complex [30,31]. Based on these previous studies, we hypothesized that STEAP4 might regulate TERT through the ROS–Nrf2 axis. First, we examined TERT expression levels in granulosa cells from PCOS patients. Both qRT-PCR and Western blotting showed elevated TERT in patients relative to controls, and STEAP4 and TERT levels were positively correlated (Figure 4B–D). To test the regulatory link directly, we examined how STEAP4 knockdown affected TERT in DHEA-treated KGN cells and found that STEAP4 depletion markedly lowered TERT at both mRNA and protein levels (Figure 4E,F). Given that ROS can regulate gene expression through the nuclear translocation of Nrf2, we asked whether STEAP4 could regulate TERT through this pathway. Western blot analysis of nuclear/cytoplasmic fractions and immunofluorescence staining showed reduced nuclear translocation of Nrf2 in STEAP4–knockdown cells (Figure 4G,H). Moreover, treatment with the oxidizing agent H_2_O_2_ (150 μM) restored TERT expression in STEAP4–depleted cells (Figure 4I). These results suggest that STEAP4 regulates TERT expression through the ROS-dependent Nrf2 signaling pathway.

### 3.5. STEAP4 Regulates TERT Expression Through HIF–1α Stability

**Figure 5:**
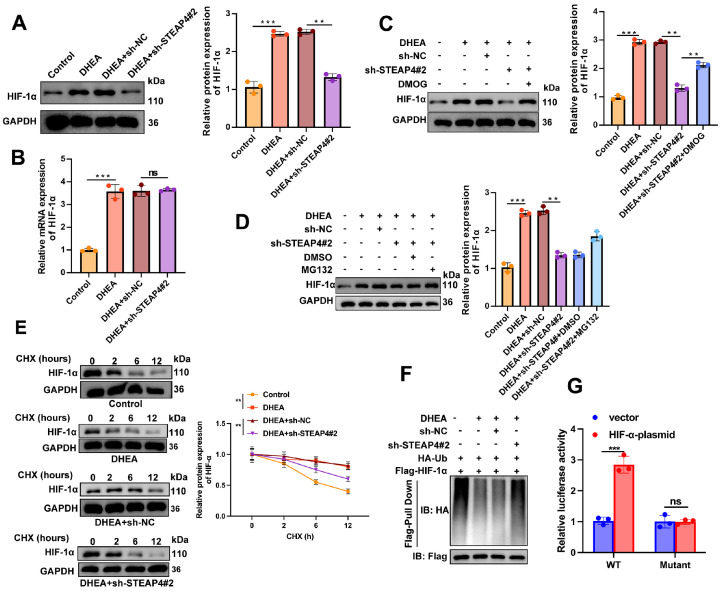
STEAP4 controls HIF–1α stability and subsequent TERT expression in DHEA–treated KGN cells. (**A**) Western blot analysis of HIF–1α protein expression levels in DHEA–treated KGN cells transfected with sh–STEAP4#2 or sh–NC. GAPDH served as the loading control. (**B**) qRT–PCR analysis of HIF–1α mRNA expression levels in DHEA–treated KGN cells after STEAP4 knockdown. GAPDH served as the internal control. (**C**) Western blot analysis of TERT protein expression levels in DHEA–treated KGN cells treated with or without the HIF–1α agonist DMOG (150 μM) for 24 h. (**D**) Western blot analysis of HIF–1α protein expression levels in STEAP4–knockdown and control DHEA-treated KGN cells treated with or without MG132 (10 μM) for 6 h. (**E**) CHX chase analysis of HIF–1α protein half-life in STEAP4–knockdown and control DHEA–treated KGN cells. (**F**) Pull down assay was used to determine HIF–1α ubiquitination levels in DHEA–treated KGN cells transfected with sh–STEAP4#2 or sh–NC. (**G**) Dual-luciferase reporter assays showing the effects of HIF–1α on TERT promoter activity in DHEA–treated KGN cells. Data are presented as mean ± SD from three independent experiments. Statistical analysis was performed using one–way ANOVA followed by Tukey’s post hoc test. ** *p* < 0.01, *** *p* < 0.001, ns, no significance.

It has been established that HIF–1α can directly induce TERT [32,33]. In addition, HIF-1α reportedly enlists NANOG as a co–activator to drive TERT transcription [34]. We therefore asked whether, in DHEA–treated KGN cells, STEAP4 governs TERT through HIF-1α. Western blotting showed that STEAP4 knockdown markedly reduced HIF-1α protein in these cells (Figure 5A). However, *HIF–1α* mRNA expression levels remained unchanged (Figure 5B), suggesting post-transcriptional regulation. Treatment with the HIF-1α agonist, dimethyloxalylglycine (DMOG; 150 μM), markedly increased TERT expression levels in STEAP4–depleted KGN cells, indicating that HIF–1α is required for TERT expression (Figure 5C). To determine whether STEAP4 regulates HIF-1α protein stability, we treated KGN cells with the proteasome inhibitor MG132. MG132 treatment rescued HIF-1α protein levels in STEAP4–depleted cells (Figure 5D). The CHX chase analysis confirmed that STEAP4 impeded the degradation of HIF–1α protein (Figure 5E). Furthermore, immunoprecipitation analysis showed increased HIF–1α ubiquitination in STEAP4–knockdown KGN cells (Figure 5F). Finally, dual–luciferase reporter assays demonstrated that HIF–1α directly activated TERT transcription in KGN cells (Figure 5G). Together, these results suggest that STEAP4–ROS signaling stabilizes HIF–1α protein to promote TERT expression.

### 3.6. Overexpression of TERT Rescues the Effects of STEAP4 Knockdown on DHEA–Induced KGN Cell Proliferation and Apoptosis

**Figure 6:**
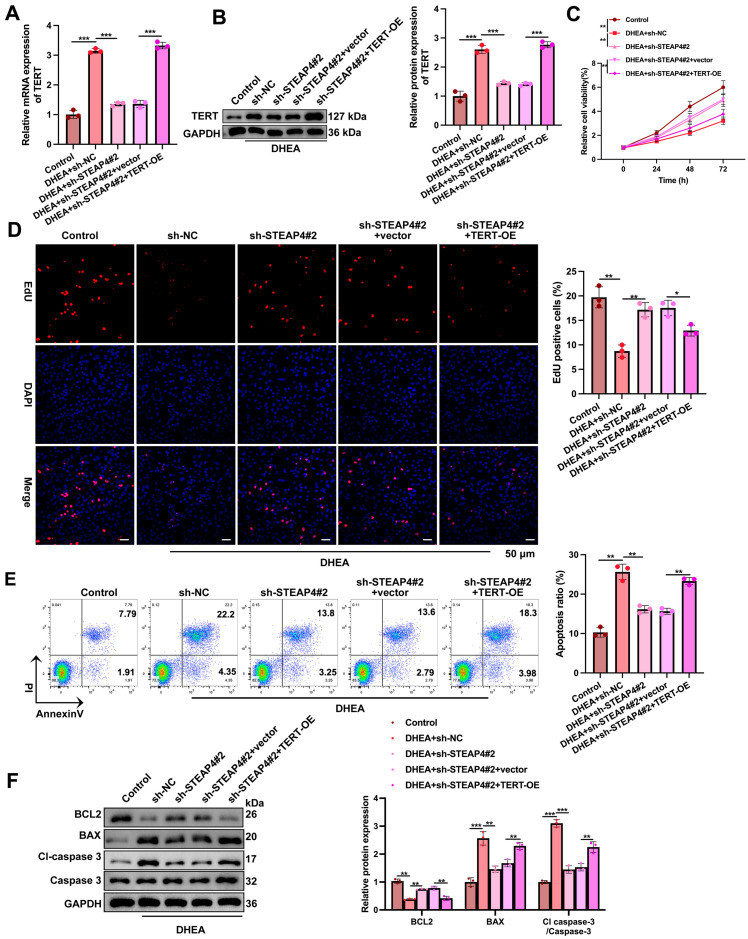
TERT overexpression reverses STEAP4 knockdown–induced phenotypes in DHEA–treated KGN cells. (**A**) qRT–PCR analysis of TERT mRNA expression levels in DHEA–treated KGN cells co–transfected with sh–STEAP4#2 and TERT overexpression plasmid or control vector. GAPDH served as the internal control. (**B**) Western blot analysis of TERT protein levels in DHEA–treated KGN cells with the indicated treatments. Representative blot (left) and quantification (right) are shown. GAPDH served as the loading control. (**C**) Cell viability was assessed using a CCK–8 assay at the indicated time points. (**D**) Cell proliferation was evaluated using the EdU incorporation assay. Representative images (left) and quantification of EdU–positive cells (right) are shown. Scale bar = 50 μm. (**E**) Flow cytometry analysis of cell apoptosis using Annexin V–FITC/PI double staining. Representative plots (left) and quantification of apoptotic cells (right) are shown. (**F**) Western blot analysis of apoptosis-related (Bax, Bcl–2, cleaved caspase–3 and caspase–3) protein expression levels in DHEA–treated KGN cells with the indicated treatments. Representative blot (left) and quantification (right) are shown. GAPDH served as the loading control. Data are presented as mean ± SD, *n* = 3. * *p* < 0.05, ** *p* < 0.01, *** *p* < 0.001.

To establish whether TERT carries out the biological actions of STEAP4 in granulosa cells, TERT was overexpressed in STEAP4–knockdown KGN cells, with overexpression efficiency verified by qRT–PCR and Western blotting (Figure 6A,B). Notably, TERT overexpression significantly reversed the increased cell viability and proliferation in STEAP4–depleted cells, as measured by CCK–8 and EdU incorporation assays (Figure 6C,D). Moreover, flow cytometry analysis showed that overexpression of TERT attenuated the decreased apoptosis induced by STEAP4 knockdown (Figure 6E). Consistently, Western blot analysis showed that TERT overexpression reversed the apoptotic protein markers in STEAP4–silenced cells, as reflected by reduced Bcl–2 alongside elevated Bax and cleaved caspase–3 (Figure 6F). These results demonstrate that TERT is a key downstream effector mediating the role of STEAP4 in granulosa cell apoptosis.

### 3.7. TERT Regulates Apoptosis in DHEA–Treated KGN Cells Through the TP53 Signaling Pathway

**Figure 7:**
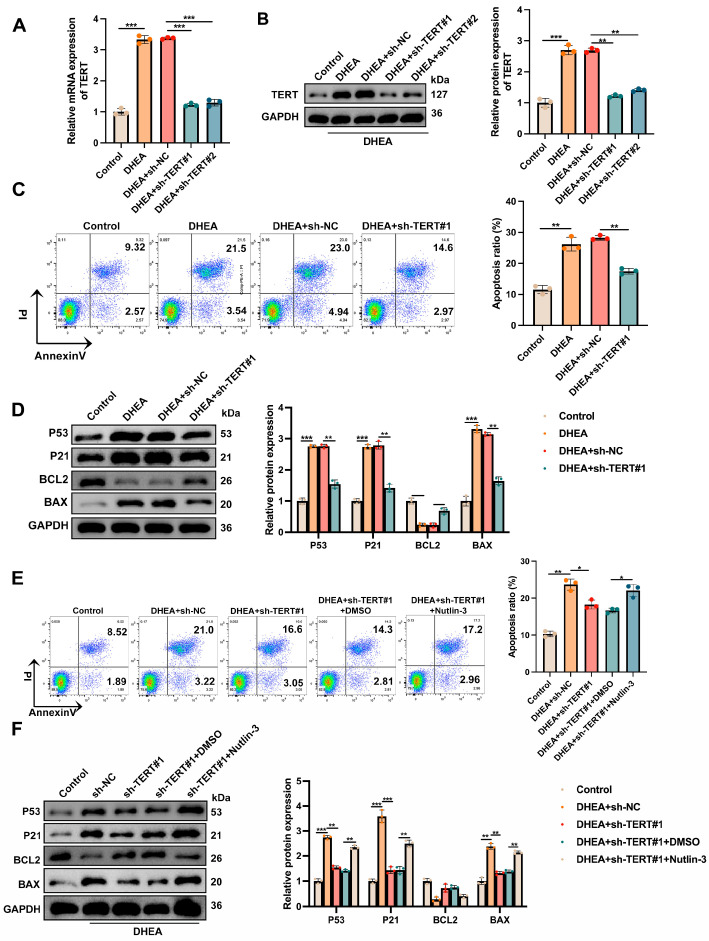
TP53 signaling mediates TERT–dependent apoptosis regulation in DHEA–treated KGN cells. (**A**) qRT–PCR analysis of TERT mRNA expression levels in DHEA–treated KGN cells transfected with sh–TERT#1/2 or sh–NC. GAPDH served as the internal control. (**B**) Western blot analysis of TERT protein expression levels in DHEA–treated KGN cells transfected with sh–TERT#1/2 or sh–NC. Representative blot (left) and quantification (right) are shown. GAPDH served as the loading control. (**C**) Flow cytometry analysis of cell apoptosis using Annexin V–FITC/PI double staining in DHEA–treated KGN cells transfected with sh–TERT#1 or sh–NC. Representative plots (left) and quantification of apoptotic cells (right) are shown. (**D**) Western blot analysis of TP53 pathway–related (TP53 and P21) and apoptosis-related (Bax and Bcl–2) protein expression levels in DHEA-treated KGN cells transfected with sh–TERT#1 or sh–NC. Representative blot (left) and quantification (right) are shown. GAPDH served as the loading control. (**E**) Flow cytometry analysis of cell apoptosis in DHEA–treated KGN cells transfected with sh–TERT#1 or sh–NC and treated with or without the small molecule MDM–2 antagonist nutlin–3 (10 μM) for 24 h. Representative plots (top) and quantification of apoptotic cells (bottom) are shown. (**F**) Western blot analysis of TP53 pathway–related (TP53 and P21) and apoptosis–related (Bax and Bcl–2) protein expression levels in KGN cells with the indicated treatments. Representative blot (top) and quantification (bottom) are shown. GAPDH served as the loading control. Data are presented as mean ± SD, *n* = 3. * *p* < 0.05, ** *p* < 0.01, *** *p* < 0.001.

Prior reports indicate that TERT modulates cellular senescence and apoptosis via the TP53 pathway across diverse diseases [35]. The TP53 pathway has, moreover, been implicated in PCOS pathogenesis and granulosa cell apoptosis [36]. We therefore examined whether TERT drives granulosa cell apoptosis through the P53 pathway. After first verifying TERT knockdown efficiency in DHEA–treated KGN cells by qRT–PCR and Western blotting (Figure 7A,B), flow cytometry showed that TERT knockdown sharply lowered apoptosis in these cells (Figure 7C). Silencing TERT markedly decreased TP53 and its downstream targets P21 and Bax while raising Bcl–2 (Figure 7D). Furthermore, treatment with the small molecule MDM2 antagonist nutlin-3 (10 μM) (which blocks the MDM2–TP53 interaction leading to increased TP53 levels) reversed the decreased apoptosis and altered expression of apoptosis-related proteins induced by TERT knockdown (Figure 7E,F). Taken together, these findings indicate that TERT drives granulosa cell apoptosis by engaging TP53–mediated signaling.

### 3.8. STEAP4 Knockdown Alleviates Systemic PCOS–like Phenotypes in Mice Through the Downregulation of TERT Expression Levels

**Figure 8:**
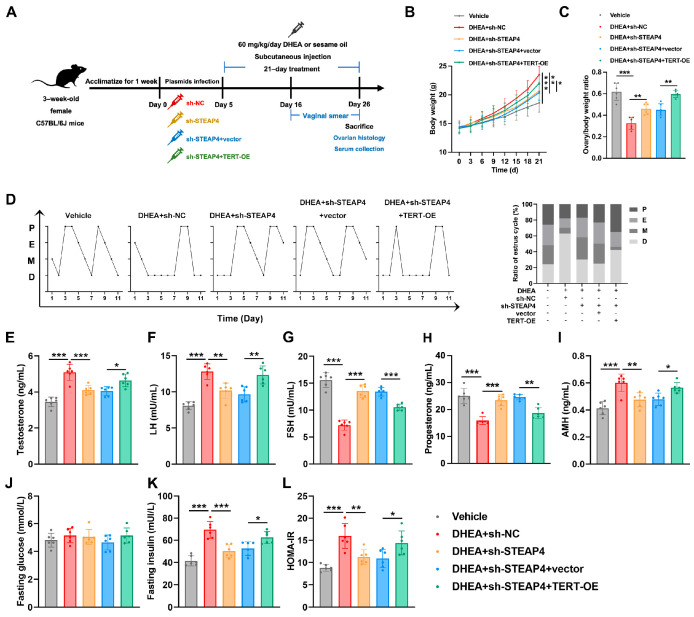
TERT overexpression reverses the protective effects of STEAP4 knockdown against PCOS–like endocrine and metabolic abnormalities in mice. (**A**) Schematic diagram of the experimental design. (**B**,**C**) Body weight curves and ovary weight (mg)/body weight (g) ratio of mice from the indicated treatment groups. (**D**) Vaginal cytology assessment of estrous cycles for 11 consecutive days. P, proestrus; E, estrus; M, metestrus; D, diestrus. Serum testosterone (**E**) and LH (**F**) levels were measured by ELISA. Serum FSH (**G**) and progesterone (**H**) levels were measured by ELISA. (**I**) Serum AMH levels were measured by ELISA. (**J**) Fasting blood glucose levels. (**K**) Fasting plasma insulin levels. (**L**) The HOMA–IR index was calculated using the following formula: [fasting glucose (mmol/L) × fasting insulin (mUI/mL)]/22.5. Data are presented as mean ± SD. * *p* < 0.05, ** *p* < 0.01, *** *p* < 0.001. *n* = 6.

The in vivo relevance of our findings was assessed using a DHEA-induced PCOS mouse model, in which STEAP4 was knocked down or TERT was overexpressed. A graphical summary of the experimental protocol is provided in Figure 8A. STEAP4 knockdown mice showed decreased body weight and an increased ovary weight/body weight ratio compared to the PCOS group, which were significantly reversed by TERT overexpression (Figure 8B,C). Analysis of vaginal smears revealed an improvement in estrous cycle disorders after STEAP4 knockdown, which was aggravated by TERT overexpression (Figure 8D). In terms of endocrine profiles, STEAP4 knockdown normalized the elevated testosterone and LH levels, as well as the decreased FSH and progesterone levels in PCOS mice, with these effects being partially reversed by TERT overexpression (Figure 8E–H). Similarly, the altered AMH levels in STEAP4–knockdown mice were restored by TERT overexpression (Figure 8I). Regarding metabolic parameters, STEAP4 interference ameliorated the elevated fasting plasma insulin levels in PCOS mice without affecting fasting blood glucose levels, while TERT overexpression partially reversed these effects, as reflected by the HOMA–IR index (Figure 8J–L). Together, these results demonstrate that TERT is essential for STEAP4–mediated regulation of systemic PCOS–like phenotypes, including reproductive function and metabolic homeostasis.

### 3.9. STEAP4 Regulates Oxidative Stress Levels via the TERT–Mediated Pathway in PCOS Mice

**Figure 9:**
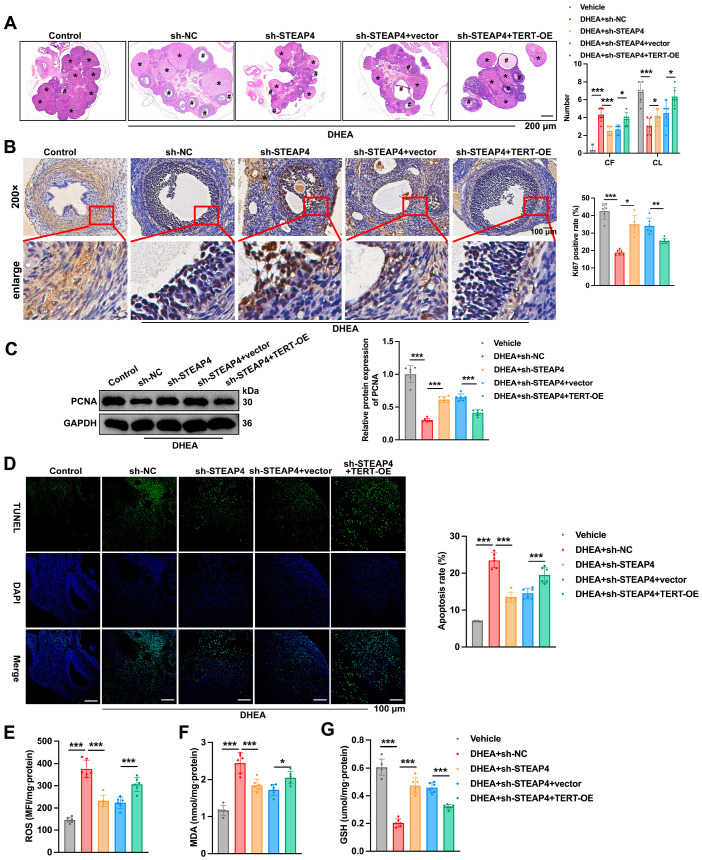
STEAP4 modulates ovarian morphology, proliferation, apoptosis, and oxidative stress via TERT in PCOS mice. (**A**) Representative H&E staining of ovarian sections (left) and quantification (right) of cystic follicles and corpora lutea. #: cystic follicles (CF). *: corpora lutea (CL). Scale bar = 200 μm. (**B**) Representative immunohistochemical staining and quantification of Ki–67 in ovarian sections. Scale bar = 200 μm. (**C**) Western blot analysis of PCNA protein expression levels in ovaries. Representative blot (top) and quantification (bottom) are shown. GAPDH served as loading control. (**D**) Representative TUNEL staining (left) and quantification (right) of apoptotic cells in ovarian sections. Scale bar = 100 μm. (**E**) ROS levels in ovarian tissues were measured by ELISA. (**F**) MDA content in ovarian tissues was measured by ELISA. (**G**) GSH levels in ovarian tissues were determined by ELISA. Data are presented as mean ± SD, *n* = 6. * *p* < 0.05, ** *p* < 0.01, *** *p* < 0.001.

We further examined the effects of STEAP4 knockdown and TERT overexpression on ovarian function and oxidative stress levels in PCOS–like mice. Histological examination showed that TERT overexpression reversed the STEAP4 knockdown–induced decrease in cystic follicles (CF) and corpora lutea (CL) (Figure 9A). Moreover, STEAP4 knockdown increased granulosa cell proliferation, as evidenced by increased Ki–67 immunostaining and PCNA protein expression levels, while decreasing cell apoptosis as shown by TUNEL staining; these effects were significantly reversed by TERT overexpression (Figure 9B–D). Notably, knockdown of STEAP4 alleviated ovarian oxidative stress, as evidenced by lower ROS and MDA alongside higher GSH, with these effects being partially counteracted by TERT overexpression (Figure 9E–G). Collectively, these findings indicate that TERT acts as a crucial downstream effector of STEAP4 in promoting PCOS progression by regulating granulosa cell proliferation, survival, and oxidative stress homeostasis.

## 4. Discussion

PCOS represents a common endocrine-gynecological condition affecting women during their reproductive years [1]. Although it ranks among the leading causes of infertility worldwide, its origins remain poorly understood, and developing more effective therapies will require a deeper insight into how the disease arises. In this study, we set out to define the link between follicular granulosa cell function and PCOS. We demonstrate that STEAP4 is elevated in the ovarian tissue of PCOS patients and that it restrains proliferation while driving apoptosis in granulosa cells. STEAP4 augments oxidative stress, which in turn promotes Nrf2 nuclear entry and stabilizes HIF–1α, leading to higher TERT levels and ensuing activation of TP53 signaling and PCOS progression. Collectively, our data implicate STEAP4 in PCOS pathogenesis and mark it as a possible therapeutic target.

To our knowledge, this is the first demonstration that STEAP4 is upregulated in granulosa cells of both PCOS patients and DHEA–induced PCOS–like mice. Upregulation of STEAP4 has previously been reported in prostate cancer, specifically in androgen and androgen receptor-expressing prostate cancer cells [15,17,37]. As STEAP4 is an androgen-dependent gene [15], the raised androgen levels in PCOS and in PCOS–like mice are the likely cause of its upregulation.

We then explored the role of STEAP4 in PCOS by knocking it down in a cell–based PCOS model, generated by exposing human granulosa–like KGN cells to the androgen DHEA. As anticipated, DHEA raised STEAP4 protein in KGN cells. STEAP4 knockdown produced a marked increase in proliferation alongside a substantial drop in apoptosis, indicating that STEAP4 is important for granulosa cell proliferation and survival. STEAP4 silencing has previously been reported to lower viability, migration and invasion and to enhance apoptosis in prostate cancer cells [37,38]. Nevertheless, to the best of our knowledge, a role for STEAP4 in governing granulosa cell proliferation and survival is reported here for the first time.

Oxidative stress is a key mediator of female reproductive disorders including PCOS [23,25]. Furthermore, oxidative damage has been shown to affect follicle quality by inducing apoptosis in granulosa cells [26]. We therefore asked whether STEAP4 takes part in regulating oxidative stress in granulosa cells. We observed that STEAP4 knockdown in DHEA–treated KGN cells substantially lowered intracellular and mitochondrial ROS along with lipid peroxidation. STEAP4 depletion further reduced MDA and raised SOD and CAT activities. Taken together, these data indicate that STEAP4 governs ROS generation and mitochondrial function in granulosa cells. Previous studies have highlighted the complex relationship between STEAP4 and oxidative stress, which appears to be dependent on the cellular environment. Under certain circumstances, STEAP4 has been shown to induce oxidative stress, thereby promoting disease progression. For example, Taylor et al. showed that diabetes-associated STEAP4 expression enhances retinal oxidative stress [39], and Jin et al. found that STEAP4 substantially raises ROS in prostate cancer cells [40]. Conversely, STEAP4 has been reported to guard against high–fat–diet–induced oxidative stress and to preserve cell viability in hepatocytes [41], while Liu et al. recently reported that overexpression of STEAP4 increased proliferation and reduced oxidative stress in benign prostatic hyperplasia [14]. Our findings suggest that STEAP4 induces oxidative stress in PCOS granulosa cells, which leads to apoptosis and subsequent follicular damage and the development of PCOS.

To elucidate how STEAP4 mediates oxidative stress and apoptosis, we performed bioinformatics analysis and identified TERT as a potential downstream effector of STEAP4. As the catalytic subunit of telomerase, TERT is best known for lengthening telomeres and forestalling replicative senescence [42]. More recent studies have uncovered non-canonical TERT functions, among them lowering cellular ROS through binding to mitochondrial DNA [43]. In the present work, TERT mRNA and protein were both confirmed to be markedly raised in granulosa cells of PCOS patients, and STEAP4 levels correlated positively with TERT in these patients. STEAP4 knockdown lowered TERT in DHEA–treated KGN cells, implying that STEAP4 acts at least in part through TERT. To our knowledge, a link between STEAP4 and TERT is described here for the first time.

Next, we showed that in our cellular model of PCOS, STEAP4–induced oxidative stress promotes TERT expression via upregulation and nuclear translocation of Nrf2. STEAP4 overexpression has previously been shown to elevate Nrf2 in human HCT116 colorectal cancer cells. Indeed, Yin et al. showed that silencing STEAP4 dampened the Nrf2–NAD(P]H:quinone oxidoreductase 1 (NQO1) pathway, thereby triggering apoptosis and autophagy and markedly shrinking xenograft colorectal tumors [44]. Their study highlights the potential of targeting STEAP4 to treat colorectal cancer and confirms that STEAP4 may mediate its effects via Nrf2, consistent with our findings.

We further found that STEAP4-induced oxidative stress promotes TERT expression through the stabilization of HIF–1α stability. A relationship between STEAP4 and HIF-1α expression in the visceral and subcutaneous adipose tissue of morbidly obese individuals has been reported previously. They observed that heightened HIF–1α, arising from tissue hypoxia and pro-inflammatory signals in obesity, led to greater visceral STEAP4 expression [45]. Since our data show HIF–1α to upregulate TERT, STEAP4 most likely controls TERT in response to oxidative stress by stabilizing HIF–1α.

At the functional level, we showed that TERT overexpression rescues the impact of STEAP4 knockdown on DHEA-induced KGN cell proliferation and apoptosis, marking TERT as a probable key downstream effector of STEAP4 in granulosa cell apoptosis. Subsequent analysis indicated that TERT governs DHEA–induced KGN cell apoptosis via TP53 signaling. These observations agree with recent reports proposing a role for TP53 signaling in PCOS pathogenesis through enhanced granulosa cell apoptosis [36,46,47].

In an in vivo DHEA–induced mouse model of PCOS, we confirmed that STEAP4 and TERT participate in disease pathogenesis. We showed that while knockdown of STEAP4 in DHEA-treated mice led to decreased body weight, increased ovary weight/body weight ratio, improvement of the estrous cycle disorder, normalized elevated testosterone and LH levels, normalized decreased FSH and progesterone levels and ameliorated elevated plasma insulin levels, overexpression of TERT reversed these effects. We additionally found that STEAP4 knockdown decreased cystic follicles and increased corpora lutea, enhanced granulosa cell proliferation, lowered apoptosis, and reduced oxidative stress, all of which were partly reversed by TERT overexpression. Our findings thus position TERT as a crucial downstream effector of STEAP4 that promotes PCOS progression by modulating granulosa cell proliferation, survival, and redox homeostasis.

On the whole, our work nominates STEAP4 as a candidate therapeutic target in PCOS. The promise of targeting STEAP4 has already been noted in other conditions, including cancers, insulin resistance, non–alcoholic fatty liver disease, and benign prostatic hyperplasia [16]. In HER2-overexpressing breast cancer, for example, disrupting the STEAP4 pathway with the iron chelator Deferiprone together with the HER2 inhibitor Lapatinib markedly curbed breast cancer cell growth in vitro [48]. A role for STEAP4 as a therapeutic target in diabetic retinopathy has likewise been described, acting through inhibition of HIF1/PKM2 signaling to limit hyperglycemia–driven retinal cell apoptosis I [49]. Our results suggest that the therapeutic reach of STEAP4 can be broadened to encompass PCOS.

This study has several limitations that should be acknowledged. First, although KGN cells are widely employed in PCOS research and preserve key granulosa cell features, their tumor origin may perturb certain cellular pathways, so confirmation in primary human granulosa cells would reinforce our conclusions. Second, the cohort was modest, comprising only 30 PCOS patients and 30 controls for the clinical samples and 6 mice per group for the animal work; larger cohorts would strengthen the statistical power and generalizability of our findings. Third, while we identified TERT as a downstream target of STEAP4 through bioinformatics analysis of public databases, comprehensive RNA sequencing of STEAP4–knockdown granulosa cells would provide a more unbiased and thorough understanding of STEAP4–regulated pathways and potentially reveal additional therapeutic targets. Fourth, although our lentiviral knockdown approach effectively reduced STEAP4 expression in mice, the development of STEAP4 knockout mice would allow for more definitive conclusions about its role in PCOS pathogenesis. Additionally, validation in other animal models, such as rats, would further strengthen the translational relevance of our findings. Finally, our study focused primarily on the ovarian manifestations of PCOS; future studies should investigate whether the STEAP4–ROS–TERT–TP53 axis contributes to the metabolic aspects of PCOS, including insulin resistance and obesity. Despite these caveats, our study supplies experimental-model evidence for STEAP4 as a candidate therapeutic target in PCOS and lays out a mechanistic framework for its part in granulosa cell dysfunction.

## 5. Conclusions

In conclusion, using KGN cells together with a DHEA-induced mouse model, our study shows that STEAP4 shapes oxidative stress and granulosa cell apoptosis through the ROS–TERT–TP53 axis. This experimental-model evidence indicates that STEAP4 may contribute to PCOS–related granulosa cell dysfunction and represents a candidate therapeutic target for future clinical study. Confirmation in primary human granulosa cells and in clinical investigations will, however, be essential to verify the translational value of these findings.

## Figures and Tables

**Table 1:** Primer sequences used for qRT–PCR analysis.

Primer	Sequence (5′–3′)	Tm (°C)	Product Size (bp)
STEAP4–F	GCGGCGAAACTTCCCTCTA	60	92
STEAP4–R	CCATAACTGAGGGAGAGGCG	60	92
TERT–F	GAGAACAAGCTGTTTGCGGG	60	139
TERT–R	AGCCATACTCAGGGACACCT	60	139
HIF-1α–F	TGAGGGGACAGGAGGATCAC	60	132
HIF-1α–R	GAGACTAGAGAGAAGCGGGC	60	132
GAPDH-F	CCATGTTCGTCATGGGTGTGA	59	153
GAPDH-R	CATGGACTGTGGTCATGAGT	59	153

**Table 2:** Primary antibodies used for Western blot analysis.

Antibody	Company (Cat. No.)	Working Concentration
Anti–STEAP4	Sigma (ABS998)	1/500
Anti–BAX	CST (2772)	1/1000
Anti–BCL2	CST (28150)	1/1000
Anti–caspase-3	Abcam (ab184787)	1/2000
Anti–cleaved caspase-3	Sigma (AB3623)	1/200
Anti–TERT	ThermoFisher (MA5-16034)	1/500
Anti–Nrf2	CST (12721)	1/1000
Anti–Lamin B1	Abcam (ab229025)	1/1000
Anti–HIF-1α	Abcam (ab179483)	1/1000
Anti–P53	Abcam (ab26)	1/1000
Anti–P21	Abcam (ab109199)	1/1000
Anti–PCNA	Abcam (ab29)	1/1000
Anti–Flag	Abcam (ab205606)	1/5000
Anti–HA	Abcam (ab9110)	1/5000
Anti–GAPDH	CST (2118)	1/1000

## Data Availability

The original contributions presented in this study are included in the article. Further inquiries can be directed to the corresponding authors.
