# Correction: Quan et al. STEAP4–Mediated ROS–TERT–TP53 Signaling Promotes Granulosa Cell Dysfunction in Experimental Models of Polycystic Ovary Syndrome. *Cells* 2026, *15*, 1220

**DOI:** 10.3390/cells15212014

**Published:** 2026-11-05

**Authors:** Xinxin Quan, Xue Xue, Huilan Ma, Lei Yang, Chen Chen, Yu Liu, Kejie Yao, Hui Yang, Rongxiang Wang, Liya Shi, Lun Suo, Qiuju Chen, Lihua Sun

**Affiliations:** 1 Department of Reproductive Medicine Center, Shanghai East Hospital, Tongji University School of Medicine, Shanghai 200120, China; shsqxx@126.com (X.Q.); twinsnow@126.com (X.X.); mahuilan88@163.com (H.M.); 2205261@tongji.edu.cn (L.Y.); breeze-cici@163.com (C.C.); liuyu555555@126.com (Y.L.); ykj11382@163.com (K.Y.); hui_yang8836@163.com (H.Y.); wangrongxiang001@126.com (R.W.);; 2 Department of Assisted Reproduction, Shanghai Ninth People’s Hospital, Shanghai Jiaotong University School of Medicine, Shanghai 200011, China

In the published publication [1], the affiliation of the authors Xinxin Quan, Xue Xue, Huilan Ma, Lei Yang, Chen Chen, Yu Liu, Kejie Yao, Hui Yang, Rongxiang Wang, Liya Shi, Lun Suo, and Lihua Sun was incorrectly stated as: 

1. Department of Cardiovascular Medicine, Shanghai East Hospital, Tongji University School of Medicine, Shanghai 200120, China;

2. Research Center for Translational Medicine, Shanghai East Hospital, Tongji University School of Medicine, Shanghai 200120, China;

3. Key Laboratory of Arrhythmias, Ministry of Education, Shanghai East Hospital, Tongji University School of Medicine, Shanghai 200120, China.

These affiliations will be replaced with the correct one:

1. Department of Reproductive Medicine Center, Shanghai East Hospital, Tongji University School of Medicine, Shanghai 200120, China.

The authors state that the scientific conclusions are unaffected. This correction was approved by the Academic Editor. The original publication has also been updated.
